# Migraine and Its Association with Hyperactivity of Cell Membranes in the Course of Latent Magnesium Deficiency—Preliminary Study of the Importance of the Latent Tetany Presence in the Migraine Pathogenesis

**DOI:** 10.3390/nu13082701

**Published:** 2021-08-05

**Authors:** Joanna Cegielska, Elżbieta Szmidt-Sałkowska, Wojciech Domitrz, Małgorzata Gaweł, Maria Radziwoń-Zaleska, Izabela Domitrz

**Affiliations:** 1 Department of Neurology, Faculty of Medical Sciences, Medical University of Warsaw, 01-809 Warsaw, Poland; joanna.cegielska@wum.edu.pl; 2 Department of Neurology, Faculty of Medicine, Medical University of Warsaw, 02-097 Warsaw, Poland; elzbieta.szmidt-salkowska@wum.edu.pl; 3 Faculty of Mathematics and Information Science, Warsaw University of Technology, 00-662 Warsaw, Poland; domitrz@mini.pw.edu.pl; 4 Department of Psychiatry, Faculty of Medicine, Medical University of Warsaw, 00-685 Warsaw, Poland; mariar@wum.edu.pl

**Keywords:** migraine, neuromuscular hyperactivity, spasmophilia, tetany, electrophysiological tetany test with ischemia and hyperventilation, CSD, NMDA receptor, magnesium deficiency

## Abstract

So far, there is no consistent and convincing theory explaining the pathogenesis of migraines. Vascular disorders, the effect of oxidative stress on neurons, and the contribution of magnesium-calcium deficiencies in triggering cortical depression and abnormal glutaminergic neurotransmission are taken into account. However, there are no reliable publications confirming the role of dietary deficits of magnesium and latent tetany as factors triggering migraine attacks. The aim of the study was to evaluate the influence of latent magnesium deficiency assessed with the electrophysiological tetany test on the course of migraine. The study included: a group of 35 patients (29 women and six men; in mean age 41 years) with migraine and a control group of 24 (17 women and seven men; in mean age 39 years) healthy volunteers. Migraine diagnosis was based on the International Headache Society criteria, 3rd edition. All patients and controls after full general and neurological examination were subjected to a standard electrophysiological ischemic tetany test. Moreover, the level of magnesium in blood serum was tested and was in the normal range in all patients. Then, the incidence of a positive tetany EMG test results in the migraine group and the results in the subgroups with and without aura were compared to the results in the control group. Moreover, the relationship between clinical markers of spasmophilia and the results of the tetany test was investigated in the migraine group. As well as the relationship between migraine frequency and tetany test results. There was no statistically significant difference in the occurrence of the electrophysiological exponent of spasmophilia between the migraine and control group. Neither correlation between the occurrence of clinical symptoms nor the frequency of migraine attacks and the results of the tetany test was stated (*p* > 0.05). However, there was an apparent statistical difference between the subgroup of migraine patients with aura in relation to the control group (*p* < 0.05). The result raises hope to find a trigger for migraine attacks of this clinical form, the more that this factor may turn out to be easy to supplement with dietary supplementation.

## 1. Introduction

Migraine is a neurological disease characterized by paroxysmal symptoms such as headaches, hypersensitivity—mainly photo- and phonophobia, and autonomic disorders—most often nausea and vomiting. During the last seventy years, many theories have been developed about the pathogenesis of this disease. Elements of the vascular concept, neuronal disorders with changes in the neurotransmitter system, disturbances in the functions of ion channels and numerous types of receptors (NMDA, AMPA, mGluR, cannabinoid, vanilloid, and PAR) as well as the triggering mechanisms and the course of the process of neurogenic inflammation are important and constantly analyzed. The contribution of genetic factors is also taken into account. Despite that many experimental studies and clinical observations have identified abnormalities in the secretion of vasoactive peptides and neurotransmitters in migraine and established the effects of drugs with different target points, still, no coherent concept has been developed to explain the triggering mechanism and course of migraine clinical symptoms in detail [1,2,3].

Among the many hypotheses regarding the mechanism of the onset of migraine attacks, disturbances in the reactivity of cerebral vessels and cerebral blood flow are considered by most researchers as primary. According to the research of Olesen et al. [4,5], during a migraine aura, moderate perfusion disorders occur, especially in the occipital cortex, insufficient to induce complete ischemia and incompatible with the supply of the brain by individual arteries [6]. They then spread to the front parts of the brain. The reduction in cerebral flow during a migraine attack has been found to be associated with the cortical spreading depression (CSD) phenomenon [7,8,9,10,11].

The self-propagating wave of transient cortical depolarization (CSD) is believed to underlie the symptoms of migraine aura and is of importance in both the pathogenesis of migraine with and without aura (in which it probably develops in clinically silent areas). However, the mechanism of its triggering in the structurally and nutritionally unaltered cerebral cortex of migraine patients remains unclear.

Presumably, most migraine attacks are triggered either by internal or external factors, the most common of which are stress and over-afferent stimulation (flicker, noise, strong odors, substances contained in certain foods). It is hypothesized that the transient destabilization of the neuronal excitatory-inhibitory balance allows internal or external factors to cause excessive activation of the cortex and creates conditions for the initiation of a wave of cortical depression. According to the results of the research by Vinogradova [12] and in earlier years by Van Harreveld [13], the triggering factor may be long-term depolarization of neurons associated with the excessive calcium-dependent release of glutamate or an increase in extracellular potassium concentration. It cannot be ruled out that both factors occur simultaneously. 

The importance of potassium disorders w CSD was already indicated in the 1990s by Olesen et al. [14]. They believed that a local increase in the extracellular concentration of potassium causes depolarization of nerve endings in the arachnoid vessels and a change in the tone of the smooth muscles of the vessels, irritation of the perivascular nerves, and neurotransmitters release.

Martens-Mantai et al. [15] studied in vitro the effect of various compounds and tetanus electrical stimulation of the entorhinal cortex on the propagation of cortical depression (CSD) between the neocortex and the hippocampus. Earlier studies on the CSD propagation pattern indicated the relative resistance of the entorhinal cortex to the propagation of the depression wave from the somatosensory cortex, 70% of the waves stopped at this medium. It turned out that the repeated phenomenon of CSD caused by the injection of a KCl solution into the somatosensory cortex and electrical stimulation of the entorhinal cortex increased the likelihood of depression reaching the hippocampus, as was the local application of an AMPA receptor blocker and a cannabinoid agonist in this part of the cortex. On the other hand, the use of the N-methyl-D-aspartate (NMDA) receptor blocker and a GABA A receptor blocker within the entorhinal cortex reduced CSD penetration into the hippocampus. Apart from the implications for the research of new anti-migraine drugs, the results of the work confirm the importance of both receptors for the initiation and enhancement or extinction of the CSD process, and thus the development of migraine symptoms.

Considering the structure of the NMDA receptor involved in the release of glutamate, magnesium-calcium disturbances may play an important role in triggering cortical depression.

One of the main functions of magnesium in the nervous system is its interaction with NMDA receptor. The magnesium ion blocks the calcium channel in the NMDA receptor. Under physiological conditions, only its removal allows the flow of sodium and calcium ions and the occurrence of excitatory glutamatergic signaling. Magnesium deficiency can therefore enhance glutamatergic neurotransmission and promote excitotoxicity and consequently lead to oxidative stress. Disturbances in the processes of oxidative phosphorylation in the form of inhibition of ATP synthesis led to the opening of calcium channels, the influx of these ions into the interior of the neuron, depolarization of the cell membrane, and extensive cortical depression, as well as the death of neurons. Reduction of metabolic processes activity was demonstrated in the area of the occipital cortex during migraine aura by magnetic resonance spectroscopy [16]. It was found that lowering the level of magnesium in the platelets of patients with migraine is associated with an increase in the level of cyclic AMP but is not related to the level of cyclic GMP, and these, in turn, are probably related to the release of neurotransmitters responsible for vasomotor disorders during the initial phase of migraine and the appearance of the CSD phenomenon [17,18]. Hypomagnesaemia may also reduce the gating of nociceptive sensations in the spinal centers, which is directly related to the pain symptoms of migraine. It is believed that abnormal glutamatergic neurotransmission is associated with neurological disorders such as migraine, chronic pain, and epilepsy [16,19,20,21,22]. 

Moreover, the results of some studies suggest that not only magnesium deficiency, but also calcium deficiency, and an incorrect ratio of ionized calcium to magnesium in the serum may affect the occurrence of migraine attacks, especially menstrual migraines [19].

In other neurological diseases changes observed in migraine (especially migraine with aura), such as disturbances in regional cerebral flow and the phenomenon of self-spreading cortical depression with the simultaneous (or sequential) activity of the brainstem, has not been found so far [20,21,22]. Thus, electrolyte disturbances may play a key role in the pathogenesis of migraines.

It is known, however, that electrolyte deficiencies, especially magnesium deficiencies, may appear overtly (i.e., in the blood serum) only in some cases. It is important to demonstrate latent deficiencies. This can be done by measuring the level of magnesium in erythrocytes (which is not readily available) or indirectly by testing the neuromuscular overactivity in an electrophysiological tetany test.

The nutritional factors that trigger migraine headache attacks have been known for years, although their mechanism of action is not always clear. In recent years, there has been growing interest in the possible role of magnesium deficiency in their initiation. The effects of its acute and chronic administration (intravenous or oral) are assessed in terms of the relief of migraine headaches and symptoms of hypersensitization [23,24].

Due to the above-mentioned hypotheses of migraine pathophysiology, the aim of this study was to assess the occurrence of neuromuscular hyperactivity (which is most often associated with hypomagnesaemia) in patients with migraines. The result of the tetany electrophysiological test with ischemia and hyperventilation was used as an indicator of this hyperreactivity. It is considered more sensitive than the assessment of serum magnesium and calcium concentrations, and is used to confirm latent tetany, i.e., one in which their values are within the normal range.

## 2. Materials and Methods

The study included a group of 35 consecutive patients from the Headache Outpatient Clinic diagnosed with migraine and a control group of 24 healthy volunteers (i.e., without headaches and other neurological ailments). The research was carried out in two sessions lasting six months each. In each session, after the qualification stage, the research described in the methodology was started immediately.

Migraine was diagnosed according to the International Headache Society (IHS) criteria, 3rd edition [25]. The study did not include people with a medical history of metabolic or hormonal disorders (including thyroid and parathyroid ones), cardiovascular diseases, cancer or other serious diseases (dementia, depression), people on elimination diets, people using drugs affecting the ions concentration, or over-the-counter supplements. All patients had normal i.e., within the laboratory norm magnesium and potassium concentration and parathyroid hormone levels in blood serum.

### 2.1. Study Groups

#### 2.1.1. Study Group with Migraine

**Figure 1:**
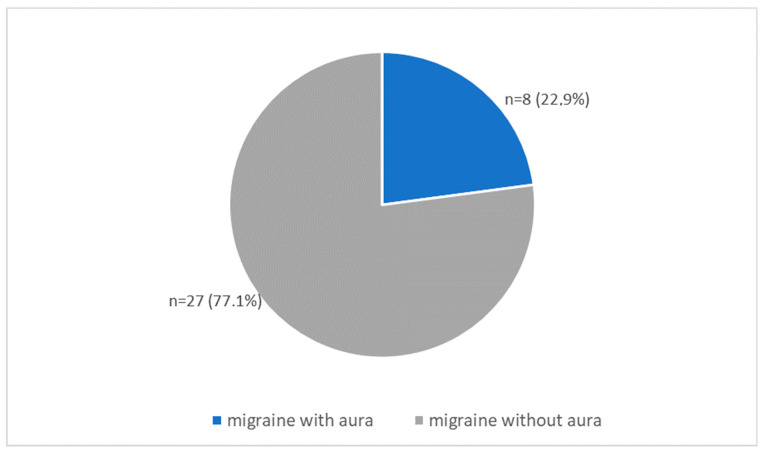
Characteristics of the type of migraine in the study group (*n* = 35).

There were 29 women (82.9%) and six men (17.1%) in the study group. Patients were between 22 to 57 years old. The study included eight patients with migraines with aura and 23 patients with migraines without aura (Figure 1).

#### 2.1.2. Control Group

The control group included 17 women (70.8%) and seven men (29.2%). The age of volunteers ranged from 23 to 61 years old.

### 2.2. Clinical Research

The frequency of the migraine attacks and other ailments (paresthesias, palpitations, vertigo, anxiety attacks, fainting) were calculated from the episodes within the last 12 months. Our patients reported that data using their notes from patient’s diaries. 

All patients and controls after full general and neurological examination (taking into account complaints and symptoms that may suggest tetany), were subjected to electrophysiological tetany test. 

Each patient and volunteer from the control group underwent a complete general and neurological examination (including complaints and symptoms suggestive of tetany), and then an electrophysiological tetany test. All of them gave their written consent to participate in the clinical trial after getting acquainted with detailed information about it. Tetany test was performed in the migraine group during the interictal periods. The test consists of an attempt to provoke the membrane hyperactivity of the motor unit with the use of controlled ischemia limited to the upper limb and additional induction of respiratory alkalosis by short-term hyperventilation. Spontaneous activity during the trial was assessed in the first dorsal interosseus muscle using disposable concentric needle electrodes (type DCN 28G × 30 mm, Myoline XP). A cuff of the sphygmomanometer was placed on the subject’s arm and held at a pressure of 10–20 mmHg higher than the previously measured systolic blood pressure, for 10 min. Hyperventilation was administered in the last 2 min of ischemia. The test was considered positive when spontaneous discharges of motor units in the multiplet sequences occurred at least five times within 5 min observation after the pressure gauge cuff was released.

The study was conducted after approval by our university Bioethics Committee and with the informed prior consent of all subjects (KB83/2015; 4 July 2015).

### 2.3. Statistical Analysis 

The statistical analysis was carried out using Statistica 13.3. *p*-values of <0.05 were considered statistically significant. Electrolytes levels and frequency of migraine episodes were treated as continuous variables. The student’s *t*-test was used to assess the differences between the independent groups for quantitative variables with separate variance estimates for age.

Pearson χ^2^ (Chi-square) and M-L χ^2^ (maximum-likelihood χ^2^) tests were used for continuous data (age, migraine severity, tetany test results). Yates χ^2^ and Fisher tests exact one-tailed and two-tailed were used due to the small size of the study groups.

## 3. Results

The demographic data and clinical characteristics of migraine, together with the results of the tetany test, are presented in Table 1. 

The demographic data, together with the results of the tetany test in the control group, are presented in Table 2.

### 3.1. Demographic Data of Both Group: Migraine vs. Control

#### 3.1.1. Sex in Migraine Group vs. Controls

In the study group (*n* = 35) there were 29 women (82.9%) and 6 men (17.1%). In the control group (*n* = 24), 17 (70.8%) and 7 (29.2%), respectively. Student’s *t*-test for age-independent samples did not reveal a statistically significant difference between the groups in terms of the number of people of both sexes.

Male 7 controls and 6 migraine *p* = 0.27.

#### 3.1.2. Age in Migraine Group vs. Controls 

**Figure 2:**
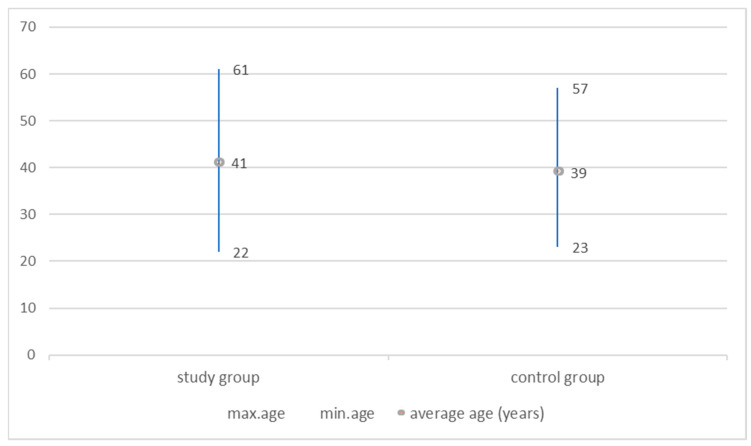
Comparison of groups by age. There was no statistically significant difference in age between the groups (*p* > 0.05).

The mean age of the patients in the study group was 41 years (SD ± 8.4), and in the control group, it was 39 years (SD ± 12.8), and it did not differ significantly between the groups. (t = 0.49; *p* = 0.63; t with separate variance estimates = 0.45 *p* = 0.65) (Figure 2).

The age in the groups is normally distributed. Kolmogorov-Smirnov (*p* > 0.2), Lilliefors (*p* > 0.2) and Shapiro-Wilk (*p* > 0.07) Tests are used.

### 3.2. Comparison of Tetany Test Results between Groups

#### 3.2.1. Tetany Test Results: Migraine Group vs. Controls 

In the migraine group, the tetany test was positive in 23 out of 35 patients (65.7%), and in the control group in 11 out of 24 patients (45.8%). Statistical analysis comparing the results of the tetany test between migraine (without and with aura) and control group showed the following values: Pearson χ^2^ = 2.30 df = 1 *p* = 0.13 and M-L χ^2^ = 2.30 df = 1 *p* = 0.13. 

**Figure 3:**
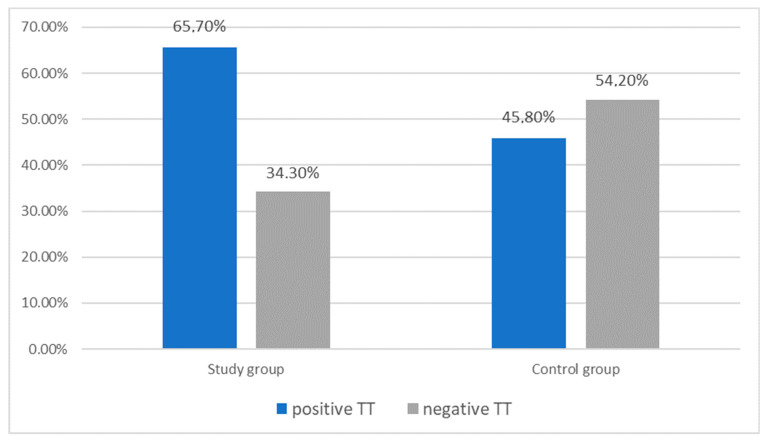
Characteristics of the study (*n* = 35) and control (*n* = 24) group according to the tetany test result. There was no statistically significant differences (*p* > 0.05).

There was no statistically significant difference in the results of the tetany test between the migraine and the control group (Figure 3).

#### 3.2.2. Tetany Test Results: Migraine Subgroup with Aura vs. without Aura vs. Control Group

In the subgroup of migraine patients with aura, 7 out of 8 persons had a positive tetany test, which constitutes 87.5% of this subgroup. In the migraine subgroup without aura, 16 out of 27 patients were positive for the test, which is 59.3%. In the control group, 11 out of 24 people (45.8%) had a positive ischemic test result.

**Figure 4:**
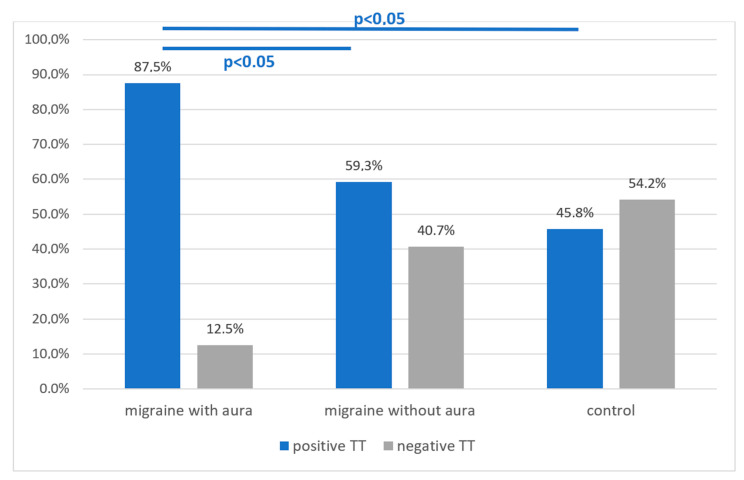
Comparison of the results of the tetany test in the migraine with aura (*n* = 8), migraine without aura (*n* = 23) subgroup and the control group (*n* = 24). A statistically significant difference was found in the migraine subgroup with aura (*p* < 0.05).

Statistical analysis showed: Pearson χ^2^ = 4.23 df = 1 *p* = 0.04 and ML χ^2^ = 4.72 df = 1 *p* = 0.03, Spearman Rank R = 0.36, t = 2.14, *p* = 0.04, and thus confirmed statistically more frequent occurrence of a positive tetany test in people with migraine with aura compared to the group without aura and to the control group (Figure 4).

### 3.3. Demographic Data of Migraine Group vs. Tetany Test

#### 3.3.1. Sex in Migraine Group vs. Tetany Test Result

A positive result of the tetany test was obtained in 19 out of 29 women (65.5%) and in 4 out of 6 men (66.7%) of the study group—no statistically significant difference. A negative result was found in 10 out of 29 women (34.5%) and 2 out of 6 men (33.3%) in this group, respectively—no statistically significant difference. (female *p* = 0.22, male *p* = 0.39).

There was no relationship between the tetany test results (positive, negative) and gender in the group of patients with migraines. 

#### 3.3.2. Sex in Migraine Subgroups vs. Tetany Test Result

Migraine without aura was diagnosed in 27 out of 35 people in the study group (77.1%), and migraine with aura in 8 out of 35 people. In both subgroups, women were clearly outnumbered: 22/27 (81.5%) and 7/8 (87.5%), respectively. Men constituted 18.5% (5/27) of the respondents in the group without aura, and 12.5% (1/8) in the group with aura. The subgroups of patients without aura and with aura did not differ statistically in terms of gender. Pearson χ^2^ = 0.03 df = 1 *p* = 0.96 and M-L χ^2^ = 0.03 df = 1 *p* = 0.96 Yates χ^2^ = 0.60 *p* = 0.44 Fisher exact one-tailed *p* = 0.22 two tailed *p* = 0.34.

#### 3.3.3. Age vs. Tetany Test in Migraine Group

The average age of patients with migraine who have had a positive tetany test result was 39 years (SD 9.7) and was similar to the average age of patients with a negative test result of 42 years (SD 4.9). 

There was no statistically significant difference in age between patients with different tetany test results (t = −0.96 *p* = 0.34; t with separate variance estimates = −1.17 *p* = 0.25).

### 3.4. Tetany Test Results vs. Clinical Data in the Migraine Group

Finally, the results of the tetany test in the study group with migraine, and various complaints reported by migraine patients that may correspond to the symptoms of tetany, collected in Table 3.

The following are the statistical analysis of these data.

#### 3.4.1. Tetany Test Results vs. the Frequency of Migraine Attacks

Among the 23 subjects in the test group with a positive tetany test, three (13.0%) had less than one migraine attack per month, two (8.7%) had two or more attacks per month and 18 (78.3%) patients had one attack per month. Among 12 patients with a negative tetany test result, the values were: 1 (8.3%), 1 (8.3%), and 10 (83.3%), respectively.

The frequency of migraine attacks did not differ statistically between patients with positive and negative tetany test results. (Pearson χ^2^ =0.18 df = 2 *p* = 0.91 and M-L χ^2^ = 0.19 df = 2 *p* = 0.91).

#### 3.4.2. Tetany Test Results vs. Incidence of General Complaints in the Migraine Group

Ailments such as: (paresthesies, cramps, faintings, fasciculations of the eyelid) were found in 15 of 23 patients (65.2%) with a positive tetany test and in 9 of 12 patients (75.0%) with a negative tetany test. 

There was no statistically significant difference in the incidence of these symptoms between patients with positive and negative tetany test (Pearson χ^2^ = 1.84 df = 2 *p* = 0.40 and M-L χ^2^ = 1.85 df = 2 *p* = 0.40).

#### 3.4.3. Tetany Test Results vs. Cardiac Arrhythmia in the Migraine Group

Palpitations occurred at different annual rates in 11 of 23 (47.8%) patients with positive tetany test and in 6 of 12 (50.0%) with a negative one. 

There was no statistically significant difference in the incidence of arrhythmias in migraine patients with positive and negative tetany test (Pearson χ^2^ = 0.015 df = 1 *p* = 0.90 and ML χ^2^ = 0.15 df = 1 *p* = 0.90 Yates χ^2^ = 0.055 *p* = 0.81 Fisher exact one-tailed *p* = 0.59 two tailed *p* = 1).

#### 3.4.4. Tetany Test Results vs. Dizziness in the Migraine Group

Dizziness was reported by 10 out of 12 (83.2%) tetany negative patients and only 10 out of 23 (43.5% of positive patients (Pearson χ^2^ = 5.11 df = 1 *p* = 0.02 and ML χ^2^ = 5.50 df = 1 *p* = 0.02 V-square df = 1 *p* = 0.03 Fisher exact one-tailed *p* = 0.03 two-tailed *p* = 0.03 Yates = 3.62 *p* = 0.57) This result was statistically significant, but the clinical significance is not. 

#### 3.4.5. Tetany Test Results vs. Sleep Disturbances in the Migraine Group

Sleep disorders were reported by 12 of 23 (52.3 %%) patients with a positive tetany test and 5 of 12 (41.7 %%) patients with a negative test.

There was no statistically significant difference in the frequency of sleep disorders in patients with positive and negative tetany test (Pearson χ^2^ = 0.34 df = 1 *p* = 0.55 and ML χ^2^ = 0.34 df = 1 *p* = 0.55 Yates χ^2^ =0.055 *p* = 0.81 Fisher exact one-tailed *p* = 0.41 two tailed *p* = 0.72).

#### 3.4.6. Tetany Test Results vs. Anxiety Attacks in the Migraine Group

Anxiety attacks occurred in 8 out of 23 (34.8%) patients with a positive tetany test and in 4 out of 12 (33.3%) patients with a negative test result. 

There was no statistically significant difference in the frequency of anxiety attacks in patients with positive and negative tetany test (Pearson χ^2^ = 0.007 df = 1 *p* = 0.93 and ML χ^2^ = 0.007 df = 1 *p* = 0.93 Yates χ^2^ = 0.084 *p* = 0.77 Fisher exact one-tailed *p* = 0.62 two tailed *p* = 1).

**Figure 5:**
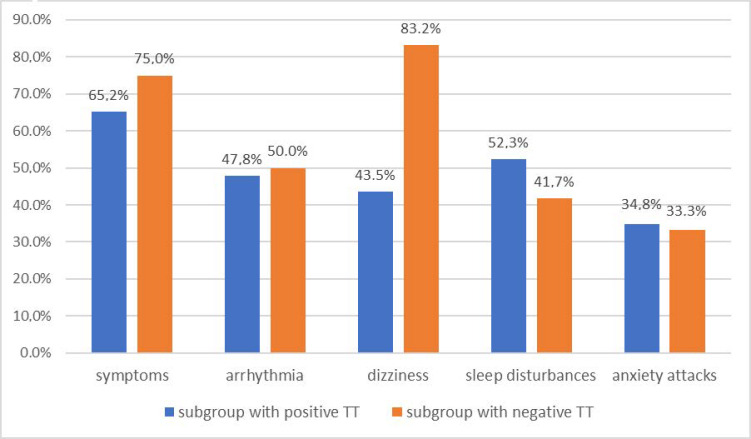
Comparison of the frequency of complaints and clinical symptoms between subgroups of migraine group with positive and negative tetany test results. There is no statistically significant differences between subgroup (*p* > 0.05), except dizziness (*p* < 0.05) which, however, is no clinically relevant.

All complaints reported by patients with a migraine that may correspond to the symptoms of tetany are summarized in Figure 5.

## 4. Discussion 

There are at least two aspects to the effects of magnesium-calcium disorders on the nervous system. Magnesium is one of the most important ions involved in cellular/mitochondrial energy changes. It takes part in numerous enzymatic reactions, and also maintains the balance of the membranes of cells, influencing its permeability and reducing the possibility of spontaneous depolarization. It affects the excitability and nerve conduction in the peripheral nervous system. Due to the correct cellular stores of this element, proper glutamatergic signaling in the central nervous system is possible, which is considered important for the protection of neurons against excitotoxicity and oxidative stress [26,27,28].

The level of magnesium measured in blood serum does not reflect its content in the internal environment of platelets, erythrocytes, or neurons and myocytes [27]. It has long been believed that an indicator of a real deficiency (sometimes in the presence of calcium-potassium disorders) is a positive result of an electrophysiological test for tetany. However, it is difficult to find the results of reliable clinical trials on this subject in the English-language literature. Short summaries are available, which do not always reflect the research methodology and scope [29,30,31]. The results of our research highlight the discrepancy between the level of magnesium in the blood serum and the results of electrophysiological tests. At the same time, they are the first attempt to find a simple indicator of the cause of a migraine and find its relationship to the lack of proper dietary magnesium supply.

The main limitation of our study is the small size of the groups. Another shortcoming of the study seems to be the extracellular measurement of magnesium concentration, which may not show real deficits of this ion. In outpatient practice, however, we cannot determine intracellular concentrations. Patients did not receive any electrolyte supplementation during the studies. It was immediately recommended to all patients with a positive tetany test result. They are still under constant medical care. The study is a preliminary report and follow-up is required. We are also thinking about modifying the method.

It has been proven that the deficiency of this macro-mineral occurs in the brain of people with migraine and has an impact on the spread of cortical depression due to the impaired function of blocking the calcium channel of the NMDA receptor (glutamatergic stimulation) and the lack of proper stimulation of the AMPA receptor. The combination of these two aspects leads to the conclusion that this simple test is useful for detecting a factor increasing the exposure of a patient diagnosed with migraine to migraine aura. It has been proved that repeated episodes of physiologically self-limiting cortical depression, in favorable conditions, e.g., hypomagnesaemia, pave the way for stimulation from the neocortex to the hippocampus through the entorhinal cortex and subcortical structures, influencing, apart from triggering the aura, the sensitization of the trigeminal autonomic system (with the effect of enhancing nociceptive sensations) and sensory secretory in the course of an attack [15,16,23,32,33,34].

The relationship between magnesium deficiency with migraine seems obvious. However, the results of several studies on the effect of intravenous administration of magnesium compounds in patients with migraines do not provide grounds for an unequivocal recommendation of this procedure. Perhaps this is due to the fact that virtually nothing is known about the correlation of magnesium disorders in migraine with the clinical course of the disease, the severity and frequency of attacks, and the age and gender index. It is difficult, both in older and recent literature, to find studies that would objectify this clinical observation based on a reliable statistical analysis of demographic and clinical data of migraine patients, also in comparison with healthy subjects. This work takes up this challenge.

It should be emphasized that the small size of the collected research groups: migraine patients and healthy volunteers, results not only from the pilot approach to the issue but also from the detailed selection of material. As can be seen from the statistical analysis, the groups were correctly selected in terms of age and gender. Moreover, people with analytically evident magnesium disorders and parathyroid hormone deficiency were excluded from the study. The study did not include people with a history of other hormonal disorders, as well as cardiological, oncological, and eating disorders that could cause non-migraine headaches, electrolyte abnormalities (e.g., anti-epileptic drugs), and damage to the peripheral nervous system, although of a different nature, but associated with disturbances of nervous excitability (e.g., drug-induced polyneuropathies).

The studies also attempted to verify the clinical symptoms that may be associated with cell hyperactivity in the course of tetania. It turned out that despite the occurrence of various general symptoms in migraine patients, such as paresthesia of the limbs and face, muscle spasms, syncope, anxiety attacks, and cardiac arrhythmias, they were not statistically related to the positive tetany test result. Therefore, this fact allows the conclusion that the mentioned symptoms do not verify tetany disorders and could only be associated with the course of migraine, including excessive sensitization known as one of the migraine phenomena.

Much of our research has shown promising results. First of all, there was a clear percentage difference in the frequency of occurrence of the electrophysiological indicator of neuromuscular hyperactivity between the migraine group and the control group (65.7% vs. 45.8%). Although this difference did not reach a statistically significant value (*p* = 0.13), the emerging tendency encourages further research on larger material. What is even more interesting, a statistically significantly higher frequency of positive results of the ischemic electrophysiological test was found in patients with migraine with aura compared to patients without aura 87.5% vs. 59.3%, and also to healthy people 87.5% vs. 45.8% (*p* < 0.05). In this case, the disproportion in the size of the compared groups is a drawback and the number of patients with aura does not reach 10, but with the available statistical methods, the correlation seems to be strong.

The above-mentioned relationship of a positive ischemic test with migraine with aura is in line with the expectations resulting from the analysis of the importance of magnesium for the maintenance of normal glutamatergic transmission via NMDA receptors (including calcium channels of this receptor). Most likely, chronic magnesium-calcium imbalance may lead to excessive glutamatergic stimulation leading to a wave of CSD cortical depression, involving the subcortical centers (mainly the entorhinal cortex with NMDAr accumulation) and the trigeminal-autonomic system. The initiation of cortical depression is not a state that occurs immediately after a decrease in magnesium levels, nor is it a state of chronic depression, so there must be other contributing factors in addition to chronic readiness for it.

## 5. Conclusions

The presented results show a relationship between migraine and aura with the occurrence of disturbances in neuromuscular excitability characteristic of latent tetany (resulting from chronic cellular ionic disturbances) demonstrated using electrophysiological tetany test with ischemia and hyperventilation. Research may contribute to the precise selection of the target group of patients responding to treatment with magnesium. However, they must be confirmed on larger material.

## Figures and Tables

**Table 1:** Demographic data, migraine clinical data and the results of the tetany test in the study group (with migraine *n* = 35). Sex: F—female, M—male. Type of migraine: A—migraine with aura, B—migraine without aura. Seizure frequency: 0—rare seizurese <1/month, 1—seizures 1/month, 2—frequent seizures >1/month. Tetany test result: 0—negative, 1—positive.

No.	Age (Year)	Sex	Type of Migraine	Seizure Frequency	Tetany Test
1	37	M	B	1	1
2	40	F	B	1	1
3	44	M	B	1	1
4	34	F	A	1	1
5	57	F	A	1	1
6	33	F	B	2	1
7	56	F	B	1	1
8	38	F	A	1	1
9	47	F	B	1	1
10	23	F	B	2	1
11	37	F	B	1	1
12	24	F	B	1	1
13	25	M	A	0	1
14	38	F	B	1	1
15	38	F	A	1	0
16	43	F	B	1	0
17	43	F	B	1	0
18	45	M	B	1	0
19	37	F	B	1	0
20	43	F	B	1	0
21	36	M	B	1	0
22	42	F	B	1	1
23	22	F	B	1	1
24	47	F	B	1	0
25	36	F	B	1	0
26	50	F	B	2	0
27	47	F	B	1	1
28	47	F	A	1	1
29	44	F	B	1	0
30	44	F	B	1	1
31	38	M	B	1	1
32	48	F	B	1	1
33	49	F	B	0	0
34	47	F	A	0	1
35	45	F	A	0	1

**Table 2:** Demographic data and the results of the tetany test in the control group (*n* = 24). Sex: F—female, M—male. Tetany test result: 0—negative, 1—positive.

No.	Age (Year)	Sex	Tetany Test
1	26	F	1
2	34	M	1
3	23	F	0
4	35	F	0
5	36	M	1
6	31	F	0
7	24	F	0
8	25	M	0
9	23	F	0
10	27	F	0
11	31	F	1
12	54	F	1
13	30	M	0
14	38	M	0
15	42	M	1
16	63	F	0
17	40	F	0
18	50	M	0
19	61	F	1
20	54	F	1
21	43	F	1
22	61	F	0
23	49	F	1
24	44	F	1

**Table 3:** Clinical and electrophysiological characteristics of the study group (*n* = 35). Clinical complains: 0—absent, +—several times a year, ++—several times a month, +++—several times a week, ++++—every day. All symptoms: 0—no complains, 1—1–2 types of ailments, 2—more than 2 types of ailments. Tetany test result: 0—negative, 1—positive.

No.	Mouth Paresthesia	Hand Paresthesia	Foot Paresthesia	Hand Cramps	Foot Cramps	Laryngospasms	Fasciculations of the Eyelide	Calf Cramps	Fainting	All Symptoms	Cardiac Arrhythmia	Dizziness	Sleep Disturbance	Anxiety Attacks	Tetany Test
1	0	++	++	0	++	0	+	++	0	2	0	0	+	0	1
2	0	0	+	0	+	0	+	0	0	2	+++	++	0	+	1
3	0	0	0	0	0	0	0	0	0	0	0	0	0	0	1
4	0	++	0	0	0	0	+	+	0	2	+	+	++	0	1
5	0	0	0	+	0	0	++	0	0	1	+	+	+++	0	1
6	0	0	0	0	0	0	++	0	0	1	++	0	++	0	1
7	0	0	0	0	0	0	0	0	0	0	0	0	0	0	1
8	0	0	++	++	++	0	++	++	++	2	++	0	++	0	1
9	0	0	0	0	0	0	0	0	0	0	0	0	0	0	1
10	0	0	0	0	0	0	0	0	0	0	0	0	0	0	1
11	0	0	0	0	0	0	++	0	0	1	++	++	++	++	1
12	0	++	++	0	++	0	++	++	0	2	+	++	0	+	1
13	0	+	0	0	0	0	+	0	0	1	0	0	0	0	1
14	++	++	++	++	++	++	++	++	++	2	0	0	++	0	1
15	0	0	+	0	0	+	+	+	0	2	+	+	0	0	0
16	0	0	0	0	0	0	+	+	+	2	+	+	++	0	0
17	0	+	0	0	0	++	+++	++	+	2	++	++	+	++	0
18	0	0	0	0	0	++	+++	++	0	2	+	++	0	0	0
19	0	+++	+++	0	0	0	++	++	0	2	0	+	+	+	0
20	+++	++++	+++	+++	+++	++++	+++	+	++++	2	++++	++++	++++	++++	0
21	0	0	0	0	0	0	0	0	0	0	++	++	0	++	0
22	0	0	0	0	0	0	0	0	0	0	++	++	++	++	1
23	0	0	0	0	+	0	++	0	0	1	0	0	++	0	1
24	0	0	0	0	+	0	0	0	0	1	0	++	0	0	0
25	0	0	0	0	0	0	++	++	0	1	0	++	0	0	0
26	0	0	0	0	0	0	0	0	0	0	0	0	0	0	0
27	0	+	+	0	0	0	+	++	0	2	0	+	+	+	1
28	0	+	0	0	0	0	+	+	0	2	+	+	+	+	1
29	0	+	0	0	0	+	0	+	0	2	0	++	++	0	0
30	0	++	0	0	0	0	++	0	0	1	+	++	+	++	1
31	0	0	+	0	0	0	0	+	0	1	0	+	0	+	1
32	0	0	0	0	0	0	0	0	0	0	0	0	0	0	1
33	0	0	0	0	0	0	0	0	0	0	0	0	0	0	0
34	0	0	0	0	0	0	0	0	0	0	+	0	0	0	1
35	0	0	0	0	0	0	0	0	0	0	0	0	0	0	1

## Data Availability

The Outpatient Clinic data.
